# Assessing genetic redundancy and diversity in Colombian cacao germplasm banks using SNP fingerprinting

**DOI:** 10.3389/fpls.2025.1632888

**Published:** 2025-07-04

**Authors:** Jaime A. Osorio-Guarín, Jhon A. Berdugo-Cely, Gina A. Garzón-Martínez, Deisy Lisseth Toloza-Moreno, Paola Delgadillo-Duran, Eliana Y. Báez-Daza, Lyndel W. Meinhardt, Sunchung Park, Dapeng Zhang, Roxana Yockteng

**Affiliations:** ^1^ Centro de Investigación Tibaitatá, Corporación Colombiana de Investigación Agropecuaria (AGROSAVIA), Mosquera, Colombia; ^2^ Centro de Investigación La Suiza, Corporación Colombiana de Investigación Agropecuaria (AGROSAVIA), Rionegro, Colombia; ^3^ Department of Agriculture, United States Department of Agriculture - Agricultural Research Service (USDA ARS) Sustainable Perennial Crops Laboratory, Beltsville, MD, United States; ^4^ Institut de Systématique, Evolution, Biodiversité-UMR-CNRS 7205, Muséum National d´Histoire Naturelle, Paris, France

**Keywords:** breeding, cacao, duplicate, germplasm banks, KASP, mislabeling, molecular marker

## Abstract

Cacao germplasm is the cornerstone of sustainable cacao production, underpinning efforts to develop high-yielding, quality-rich, and climate-resilient varieties. This study aimed to evaluate the mislabeling, the genetic redundancy, and diversity of two cacao germplasm banks maintained at the Palmira and La Suiza research centers of the Corporación Colombiana de Investigación Agropecuaria (AGROSAVIA), from Colombia. We genotyped 4,653 cacao trees from these collections, and after applying quality control filters, a final set of 77 SNP markers was used for all subsequent analyses. Our results revealed that both collections exhibit a similar pattern of genetic diversity. However, a medium rate of mislabeling (12.4%) and high genetic redundancy (53.1%) were detected, likely due to errors in collecting, introduction, pre-planting labeling, and the use of rootstocks. To optimize the evaluation conserved cacao germplasm, we defined core collections independently, which comprise 246 and 190 samples for Palmira and La Suiza, respectively. This research demonstrates the importance of maintaining a well-classified cacao collection with minimal genetic redundancy, thereby improving accuracy and reducing maintenance costs. This will not only enhance conservation efforts but also enrich the genetic diversity of the collection.

## 1 Introduction

Cacao (*Theobroma cacao* L.) is an economically important arboreal species native to the Amazon basin that produces beans of great economic importance, as they are the raw material to produce chocolate and other derivatives used in cosmetics and food industries. This plant species is predominantly a self-incompatible species with hermaphroditic flowers, requiring cross-pollination for successful fertilization (Bartley, 2005). It is grown in the tropical regions of West Africa, Central and South America, and Southeast Asia, accounting for 76.4%, 17.7%, and 6% of the world’s cocoa bean production, respectively (Bartley, 2005; ICCO, 2024). Today, cacao plantations are a vital source of income for millions of smallholder farmers in tropical regions, contributing significantly to global trade and the economy of producing countries (Kongor et al., 2024). Beyond its economic value, *T. cacao* harbors rich genetic diversity, making it a subject of scientific interest for breeding programs to enhance crop productivity, disease resistance, and quality traits in cocoa beans.

Cacao yield can be increased by improving both crop management practices and the genetic gain of cultivars, which is possible due to the genetic diversity of the crop (Rodriguez-Medina et al., 2019). Based on molecular markers, Motamayor et al. (2008) divided the genetic diversity of cacao into ten genetic groups: Amelonado, Contamana, Criollo, Curaray, Guiana, Iquitos, Marañon, Nacional, Nanay, and Purús. Since then, additional genetic groups have been reported in Ecuador and Colombia (Argout et al., 2023), Bolivia (Zhang et al., 2012), and Peru (Arévalo-Gardini et al., 2023; Zhang et al., 2023). To conserve this genetic diversity, the International Cocoa Germplasm Database (ICGD) has registered information on around 24,000 cacao accessions, including wild and improved materials (Daymond and Bekele, 2022). Currently, a total of 54 gene banks are listed across 39 different countries; however, the largest collections are conserved primarily in national research institutes. For example, Brazil preserves clones collected in the Brazilian lower Amazon and the local series CEPEC, Seleçâo Instituto de Cacao (SIC), and Seleçâo Instituto Agronómico Lesto (SIAL) at the Comissão Executiva do Plano da Lavoura Cacaueira/Centro de Pesquisas do Cacau (CEPEC/CEPLAC). Costa Rica safeguards accessions from the local series Centro Cacao (CC), United Fruit Co (UF), Programa de Mejoramiento de Cultivos Tropicales (PMCT), Area de Recursos Fitogenéticos (ARF), and Trinidad & Tobago preserves the largest collection in the world (Kodoth, 2021; López-Hernández et al., 2021; Daymond and Bekele, 2022).

In Colombia, of the total plant accessions maintained under *ex situ* conditions in germplasm banks, 70% are administered by the Corporación Colombiana de Investigación Agropecuaria (AGROSAVIA). The first germplasm collection in the country was established in 1960 in the experimental Palmira station in Valle del Cauca department, which served as the headquarters of the national cacao research program under the no longer existing Department of Agricultural Research (DIA) (Rodriguez-Medina et al., 2019). In 1995, the collection was duplicated at AGROSAVIA’s La Suiza research center in Santander department and expanded by the acquisition of cacao genetic materials from international sources, as well as collections made throughout Colombia, including 400 wild-type accessions. AGROSAVIA preserved this collection at both Palmira and La Suiza, following a homologation process to standardize the number of accessions between the two sites (Rodriguez-Medina et al., 2019).

These collections contain accessions dedicated to the conservation of genetic diversity and other accessions that serve as a working collection. Both represent a critical resource for addressing key agricultural challenges for Colombian cocoa farmers, including low yield, heavy metal accumulation such as cadmium and, high incidence of diseases such as frosty pod rot (FPR), witches’ broom (WBD), black pod, low pollination rates. To address these challenges and contribute to sustainable cocoa production and support improved farmer livelihoods, AGROSAVIA’s national cacao breeding program leverages these collections through pre-breeding and recurrent selection strategies (Rodríguez-Medina et al., 2023). Phenotypic evaluations have yielded important insights into valuable agronomic traits. For example, Osorio-Guarín et al. (2020) evaluated genotypes for four key traits: number of healthy pods (as a proxy for productivity), resistance to FPRD and WBD, measured using the area under the disease progress curve (AUDPC) for infected pods, flower cushion broom, and deformed branches. Among the genotypes evaluated, GS-29, FCM-39, and EET-8 showed superior productivity. SUI-72, CRICF-13, EBC-06, and EBC-09 demonstrated strong resistance to FPRD, while SCC-85, SCC-86, SUI-99, EET-377, UF-273, and FCM-19 were notable for their tolerance to WBD. In addition, the development of TCS 01, TCS 06, TCS 13, and TCS 19 varieties by AGROSAVIA represents a major outcome of long-term genetic improvement initiatives. These cultivars, derived from the national germplasm collection, were selected for their outstanding agronomic performance and productivity (Agudelo-Castañeda et al., 2017; Suárez et al., 2022).

Ensuring the integrity of plant germplasm collections is critical for preserving genetic diversity and supporting agricultural research and breeding efforts. Identifying and removing duplicates within germplasm banks is therefore crucial to enhance the accuracy and efficiency of these collections (Singh et al., 2019). Duplicates occupy valuable space and increase the cost and labor required for conservation. Consequently, implementing robust methodologies to detect duplicates is essential for safeguarding genetic resources, reducing costs, and optimizing their use in breeding programs and scientific research (van Hintum, 2000).

The characterization of cacao germplasm can be assessed using morphological or molecular diversity analyses (Aikpokpodion et al., 2010; Ballesteros and Lagos, 2016; Cosme et al., 2016; Mahabir et al., 2017; Osorio-Guarín et al., 2017; Ramos Ospino et al., 2020; Wang et al., 2020; Gutiérrez et al., 2021). Each approach offers distinct advantages and limitations that have been widely discussed (Bunjkar et al., 2024). Morphological characterization efficiency is often limited by the time and effort required to obtain results. This process is even more complex when genotype by environment interactions (G x E) effects are considered, as multi-location trials are necessary, increasing both costs and logistics (Temesgen et al., 2021; Yadesa, 2022). In contrast, molecular analysis can complement traditional approaches in identifying duplications, as molecular markers provide genome-wide coverage and are unaffected by environmental factors. In addition, molecular markers have been widely used to study the population structure and genetic diversity of germplasm collections. Nowadays, they can be identified in large numbers and used to compare divergence between genotypes, estimate their relationship, and finally help to speed up breeding programs (Van Treuren and Van Hintum, 2005).

Various molecular markers, especially simple sequence repeats (SSRs), also known as microsatellite markers, have been widely used as an international standard for cacao DNA fingerprinting and germplasm screening (Borrone et al., 2007; Zhang et al., 2009; Aikpokpodion et al., 2010; Irish et al., 2010). However, SSR markers have disadvantages, due to their high cost, labor-intensive protocols, and the difficulty of cross-platform data comparison (Livingstone et al., 2011). In contrast, single nucleotide polymorphism (SNP) markers have become the most commonly used marker type in large-scale genomics due to their cost-effectiveness, automation potential, and suitability for high-throughput analysis (Mammadov et al., 2012; Tripodi, 2023). Among these, the KASP (Kompetitive allele-specific PCR) platform has emerged as a preferred SNP genotyping method, offering low error rates, scalability, and a significant reduction in cost per data point (Semagn et al., 2014). SNP genotyping has proven to be a highly effective tool for genebank management in Africa (Padi et al., 2015; Olasupo et al., 2018; Li et al., 2021; Asare Bediako et al., 2025), the Americas (Cosme et al., 2016; Osorio-Guarín et al., 2017; Lindo et al., 2018; Mahabir et al., 2020), and the Asia-Pacific region (Lukman et al., 2014; Wang et al., 2020; Dillon et al., 2024).

At AGROSAVIA, a previous effort used 96 SNP markers to assess the genetic diversity analysis and population structure within the cacao germplasm bank and was mainly focused on identifying four main subpopulations (Osorio-Guarín et al., 2017). However, that analysis did not include all the accessions or multiple samples per accession. In the present study, we applied SNP markers using KASP technology to identify duplicates and mislabeled accessions in AGROSAVIA’s cacao germplasm banks in Palmira and La Suiza. We also evaluated the genetic structure of accessions. The generated information allowed curators to more accurately detect redundant genetic material, thereby enhancing the efficiency and accuracy of cacao germplasm conservation.

## 2 Materials and methods

### 2.1 Genetic materials

The Colombian cacao germplasm collection, managed by the Corporación Colombiana de Investigación Agropecuaria – AGROSAVIA (Colombia), is maintained *in vivo* at the research center Palmira (3°30′41″N 76°19′19″W) located at 1,001 m.a.s.l, 23°C mean temperature and mean annual rainfall of 1,017 mm, and at the research center La Suiza (7°22′12″N, 73°11′39″W) located at 298 m.a.s.l, 30°C mean temperature and mean annual rainfall of 1,800 mm. The germplasm banks are composed of accessions for conservation purposes as well as additional accessions from working collections. An accession is defined as a distinct and uniquely identifiable sample of a cultivar, breeding line, or population. Each accession is typically represented by multiple tree (up to five) samples propagated vegetatively, by grafting or rootstock, which are considered clones. The two germplasm banks were selected because La Suiza is located in one of the Colombia’s main cacao-producing regions, while Palmira offers contrasting climatic conditions and the possibility to evaluate distinct traits to La Suiza.

In this study, we sampled adult leaves of each tree of the 456 accessions in Palmira and 390 accessions in La Suiza, for a total of 4,653 samples collected (**Supplementary Table 1**). These samples were stored in hermetically sealed bags with approximately 100 grams of silica gel for transportation to the molecular laboratory at Tibaitatá (4°41′45″N 74°12′12″W) research center of AGROSAVIA. Plant material was washed with water, rinsed with 75% ethanol, and dried with a paper towel. Six to eight leaf disks from each tree were punched and loaded into the 96-well BioArk sampling kits from LGC Biosearch Technologies (https://www.biosearchtech.com/). The sample kits were shipped to the USDA-ARS, Beltsville Agricultural Research Center.

### 2.2 DNA extraction and genotyping

Genomic DNA was extracted by the LGC Genomics services using the sbeadex mini plant kit (LGC Genomics) following the manufacturer’s instructions. We selected 96 SNP markers from a larger database containing over 1,000 SNPs, previously published on cacao research (Allegre et al., 2012; Gutiérrez et al., 2021). The selection criteria for the SNP panel included: distributed across the 10 cacao linkage groups, call rate, minor allele frequency (MAF), and Shannon’s information index. The SNP and the flanking sequences were submitted to LGC Biosearch Technologies for genotyping using a Kompetitive allele-specific array (KASP) (**Supplementary Table 2**). This method is based on a competitive allele-specific dual FRET-based assay (Cuppen, 2007). Genotype calling was performed using the SNPviewer software (LGC Biosearch Technologies, Hoddesdon, UK).

### 2.3 Genetic diversity analyses

The quality control of raw data for the 96 SNPs was performed using the assurance module of the SNP Variation Suite software v8 (SVS8; Golden Helix Inc., Bozeman, Montana). SNPs with a call rate greater than 90% were retained in subsequent analyses. We generated a genotype accumulation using the *genotype_curve* function of the package poppr in R v4.3.0 (Kamvar et al., 2014) to determine the minimum number of markers necessary to discriminate between individuals in a population.

Descriptive statistics, including the Shannon information index (I), observed heterozygosity (H_O_), and expected heterozygosity (H_E_), were calculated using the whole collection, after filtering genetically identical individuals, and for the core collections. The fixation indexes (F_IT_, F_ST_, and F_IS_) per marker were also calculated. A principal component analysis (PCA) was conducted to elucidate genetic relationships among accessions using Nei’s genetic distance matrix. Finally, molecular analysis of variance (AMOVA) was calculated to assess the amount of genetic variation within and among populations. All the analyses were carried out using GenAlEx v6.503 software (Peakall and Smouse, 2012).

### 2.4 Assessing plot heterogeneity, mislabeling, and genetic redundancy

The probability that two individuals share the same multilocus genotype (MLG -** a** unique combination of alleles observed at multiple genetic loci) is commonly referred to as the probability of identity (PID). The probability of identity among siblings (PID-sib) estimates the likelihood that two randomly selected siblings from a population share the same MLG. Both probabilities were calculated using GenAlEx 6.503 (Peakall and Smouse, 2006, 2012) with established formulas (Waits et al., 2001).

Samples with the same accession name and grouped within the same plot (a defined field area where a plant or group of plants are grown), but showing non-matching SNP patterns, were considered cases of mislabeling. These were identified through multilocus matching using the software GenAlEx 6.503 (Peakall and Smouse, 2006, 2012), following the methodology described in previous studies (Padi et al., 2015; Zhang and Motilal, 2016; Olasupo et al., 2018). A plot heterogeneity was declared when a plot had more than one type of SNP patterns. Individuals with different accession names but with fully matching SNP patterns were considered duplicates. The identification of duplicates was based on the identification of synonymous groups (sets of samples that have the same MLGs), having a maximum genetic distance threshold of 0.05. using the functions *mlg.filter* and *mlg.id* in the poppr package (Kamvar et al., 2014).

### 2.5 Population structure analysis

After examining duplicates, an assignment test was applied to infer the ancestry (hybrids or ancestral forms) of the cacao accessions. For this purpose, a model-based clustering method was implemented in the Structure software v2.3.4 (Pritchard et al., 2000). Data from individuals belonging to the ten cacao reference groups (Motamayor et al., 2008) were included to analyze their ancestral contribution to AGROSAVIA’s germplasm. The detailed list of the 10 reference groups was provided in **Supplementary Table 3**. The sample size of each reference group was brought up to 200 using the simulation procedure implemented in the computer program ONCOR (Kalinowski et al., 2007). The simulated populations were then analyzed together with the AGROSAVIA collection. The analysis used a mixed model with a cluster number (K value) of 10, corresponding to the possible genetic groups of cacao present in the AGROSAVIA accessions. Ten independent runs were performed using 100,000 iterations after a burn-in period of 50,000. The run with the highest value of Ln Pr (X|K) of the ten runs was chosen and presented in a bar diagram. The Q value represented each germplasm group’s ancestral contribution.

### 2.6 Core collection

The core collection was identified using Core Hunter 3 software (De Beukelaer et al., 2018), applying a sampling intensity of 20% to the dataset after removing duplicate samples. The algorithm optimized genetic diversity by maximizing Modified Rogers’ distance among selected accessions, ensuring the core subset captures the broad genetic variation of the collection. The heterozygosity of the core collection and of the complete collection were compared to confirm that represents the overall genetic diversity.

## 3 Results

### 3.1 Genotyping and genetic diversity analysis

In total, 4,653 trees from 553 accessions were genotyped. The two collections shared 304 accessions, with 162 unique to Palmira and 87 unique to La Suiza. Markers with a call rate below 90% were removed from the initial dataset with 96 SNPs, leaving a final dataset of 77 SNPs for 2,597 accessions from Palmira and 2,056 from La Suiza (**Supplementary Tables 1**, **4**).

**Figure 1:**
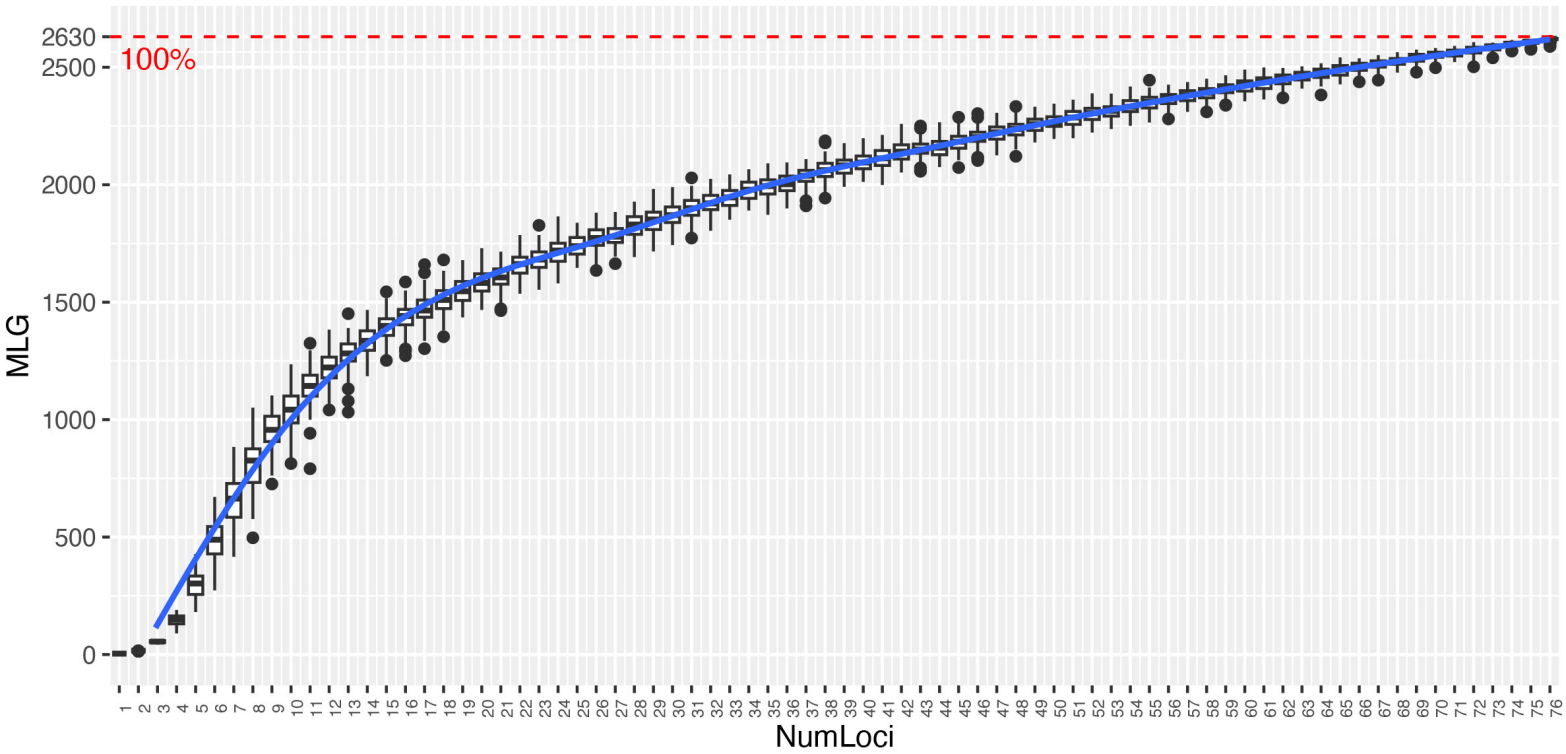
Genotype accumulation curve for 4,653 cacao samples genotyped at 77 loci. The horizontal axis represents the number of loci, and the vertical axis shows the number of unique multilocus genotypes (MLGs) observed. The red dashed line represents 100% of the observed unique multilocus genotypes.

The genotype accumulation curve showed a tendency to reach a plateau and had a greatly decreased variance with 77 SNPs, indicating that there were enough markers to identify 100% of the 2,630 MLGs (**Figure 1**). The diversity indices were similar in both collections (**Table 1**). The I index quantifies the amount of genetic diversity in a population based on the frequencies of alleles observed across the genetic loci analyzed. Presented a mean value of 0.588 (Palmira = 0.593 and La Suiza = 0.583). Among the 77 SNP markers, the H_O_ ranged from 0.106 to 0.662, with an average of 0.399 for the two germplasm banks. The Palmira collection showed a mean value of 0.394, and La Suiza had a mean value of 0.404. The H_E_ ranged from 0.199 to 0.500, with an average of 0.404. The Palmira collection presented a mean value of 0.408, and La Suiza had a mean value of 0.4. After removing the duplicate samples (2,472), the collection consisted of 2,181 samples, with the I and He indexes slightly higher, while the Ho index was lower.

**Table 1:** Comparison of prior, post fltering of duplicates and for core collection of the information index (I), observed heterozygosity (H_O_), and expected heterozygosity (H_E_) in the Colombian cacao collections maintained in Palmira and La Suiza research centers.

Collection	Statistics	Whole collection (4,653 samples)			After filtering duplicates (2,181 samples)			Core collection (La Suiza190 samples) Palmira (246 samples)		
		I	H_O_	H_E_	I	H_O_	H_E_	I	H_O_	H_E_
Palmira	Mean	0.593	0.394	0.408	0.602	0.377	0.416	0.619	0.418	0.431
	SE*	0.013	0.016	0.012	0.012	0.014	0.011	0.010	0.010	0.009
La Suiza	Mean	0.583	0.404	0.4	0.585	0.354	0.402	0.606	0.421	0.419
	SE*	0.015	0.019	0.013	0.015	0.015	0.013	0.011	0.011	0.010
Total	Mean	0.588	0.399	0.404	0.594	0.366	0.409			
	SE*	0.01	0.012	0.009	0.009	0.010	0.008			

*SE, Standard error.

For F statistics, most loci showed an excess of heterozygosity (**Table 2**), and the F_ST_ index indicated a low level of differentiation between the two germplasm banks. This is confirmed by the fact that F_IS_ and F_IT_ have almost the same value. We obtained a mean value of 0.041 for F_IS_, 0.042 for F_IT_, and 0.001 for F_ST._ AMOVA was employed to assess the distribution of the observed genetic variance between the two collections and showed that genetic variance within accessions contributed 100% to genetic diversity (**Table 3**), while the variance among populations was 0% of the total variance. This demonstrates that the observed genetic variations primarily arise from variation among individuals within each germplasm bank rather than between different germplasm banks.

**Table 2:** F-statistics (F_IS_, F_IT_, and F_ST_) per locus and mean values.

Locus	F_IS_	F_IT_	F_ST_
TcSNP13	-0.020	-0.020	0.000
TcSNP32	-0.148	-0.147	0.001
TcSNP64	-0.084	-0.083	0.000
TcSNP131	-0.028	-0.028	0.000
TcSNP139	-0.166	-0.166	0.000
TcSNP141	-0.064	-0.064	0.000
TcSNP143	-0.172	-0.171	0.001
TcSNP144	-0.102	-0.102	0.000
TcSNP148	-0.156	-0.156	0.000
TcSNP150	-0.138	-0.137	0.000
TcSNP154	-0.036	-0.034	0.002
TcSNP173	-0.165	-0.165	0.000
TcSNP226	-0.060	-0.060	0.000
TcSNP230	0.032	0.033	0.000
TcSNP242	-0.139	-0.139	0.000
TcSNP281	0.022	0.026	0.004
TcSNP290	0.129	0.129	0.001
TcSNP309	-0.121	-0.121	0.001
TcSNP339	0.071	0.072	0.002
TcSNP341	-0.139	-0.139	0.000
TcSNP363	-0.109	-0.109	0.000
TcSNP372	-0.037	-0.036	0.002
TcSNP380	-0.137	-0.137	0.000
TcSNP414	0.083	0.084	0.001
TcSNP429	0.289	0.289	0.000
TcSNP519	-0.096	-0.095	0.001
TcSNP522	-0.132	-0.131	0.001
TcSNP534	-0.198	-0.198	0.000
TcSNP546	-0.043	-0.043	0.000
TcSNP560	-0.075	-0.075	0.000
TcSNP577	0.220	0.220	0.000
TcSNP591	0.212	0.214	0.002
TcSNP619	-0.061	-0.060	0.001
TcSNP636	0.021	0.021	0.000
TcSNP642	0.038	0.039	0.000
TcSNP645	-0.029	-0.029	0.000
TcSNP703	-0.109	-0.107	0.002
TcSNP723	0.087	0.088	0.001
TcSNP953	0.088	0.088	0.000
TcSNP994	-0.048	-0.047	0.001
TcSNP1060	0.018	0.018	0.001
Tcm001s08353112	0.193	0.194	0.001
Tcm001s36937466	0.001	0.001	0.000
Tcm001s37350335	0.201	0.204	0.004
Tcm002s00846551	0.385	0.386	0.002
Tcm002s07831310	0.203	0.206	0.004
Tcm002s10122518	0.442	0.442	0.000
Tcm002s34592845	-0.023	-0.023	0.000
Tcm002s35727800	0.141	0.142	0.001
Tcm003s32803814	0.346	0.348	0.002
Tcm004s02695191	-0.085	-0.084	0.001
Tcm004s04980962	0.127	0.128	0.000
Tcm004s20311304	-0.241	-0.241	0.000
Tcm004s23094642	0.122	0.125	0.004
Tcm004s25603617	0.463	0.465	0.003
Tcm005s05086606	-0.141	-0.138	0.002
Tcm005s07951625	0.287	0.289	0.003
Tcm005s30523742	-0.095	-0.094	0.000
Tcm005s31040891	-0.109	-0.109	0.000
Tcm005s31319407	-0.086	-0.086	0.000
Tcm005s38962849	0.203	0.203	0.001
Tcm006s16820907	0.231	0.232	0.001
Tcm006s22513846	0.472	0.473	0.002
Tcm006s25228227	0.021	0.021	0.000
Tcm006s25344190	0.044	0.045	0.000
Tcm007s00632521	0.010	0.010	0.000
Tcm007s04235015	0.086	0.086	0.000
Tcm007s04402504	-0.011	-0.010	0.001
Tcm007s04810899	0.164	0.165	0.001
Tcm008s00343038	0.165	0.165	0.000
Tcm008s00740874	0.274	0.274	0.000
Tcm008s03821013	0.346	0.346	0.000
Tcm008s05744556	0.155	0.158	0.003
Tcm009s03673797	0.147	0.147	0.000
Tcm009s29674603	-0.151	-0.150	0.000
Tcm010s02045420	0.265	0.266	0.002
Tcm010s03831523	0.101	0.101	0.000
Mean	0.041	0.042	0.001
SE	0.019	0.019	0.000

**Table 3:** AMOVA analysis for the 4,653 cacao genotypes based on 77 SNP markers.

Source	df	SS	MS	EV	Percentage (%)
Among Populations	1	153.547	153.547	0.051	0
Within Populations	4651	170659.350	36.693	36.693	100
Total	4652	170812.897		36.744	100

df, Degrees of freedom; SS, Sum of squares; MS, Mean squares; EV, Estimated variance.

**Figure 2:**
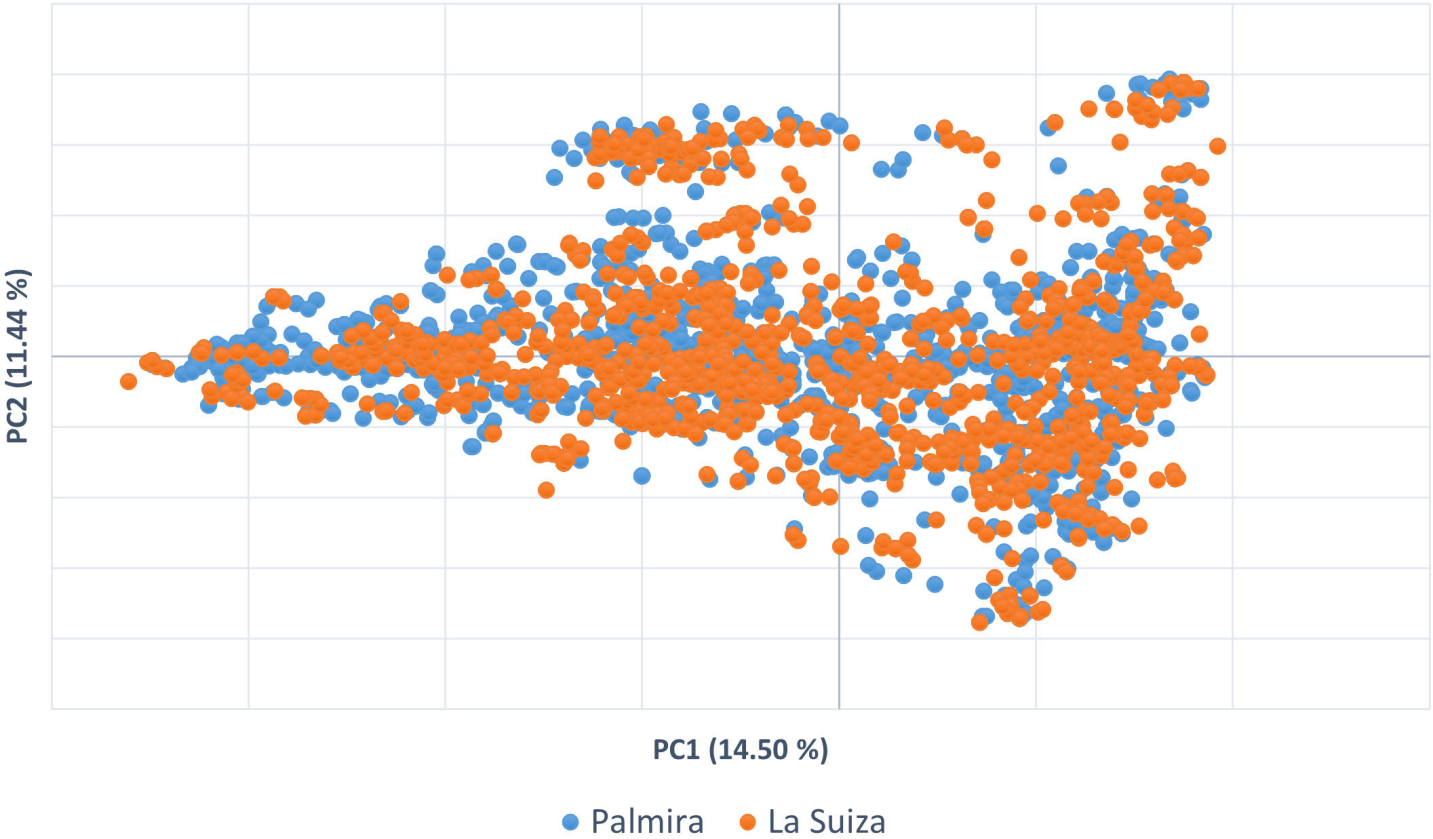
Principal component analysis (PCA) in two coordinates to visualize the distribution of the cacao germplasm accessions of the two collections.

To visualize the relationships between the accessions of *T. cacao*, a PCA was done from Nei’s genetic distance matrix. The first two PCA coordinates accounted for 26% of the variability (**Figure 2**). In the graph, two general clustering trends can be observed.

### 3.2 Assessment of plot heterogeneity, mislabeling, and genetic redundancy

The combined PID-sib of the 77 SNP panel was 1.203 x 10–^15^ and ranged from 5.95 x 10–^1^ to 7.3 x 10^-1^. In addition, the PID based on the combination of 77 SNP loci was 1.479 x 10^-29^, indicating an extremely low probability of two unrelated individuals sharing the MGL by chance (**Table 4**).

**Table 4:** Probability of identity (PID) and probability of identity among siblings (PID-sib_)_ for each of the 77 loci.

SNP	PID	PID-sib
TcSNP013	4.3E^-01^	6.5E^-01^
TcSNP032	3.9E^-01^	6.1E^-01^
TcSNP064	3.8E^-01^	5.9E^-01^
TcSNP131	4.9E^-01^	7.0E^-01^
TcSNP139	3.9E^-01^	6.1E^-01^
TcSNP141	6.5E^-01^	8.1E^-01^
TcSNP143	3.9E^-01^	6.1E^-01^
TcSNP144	4.4E^-01^	6.6E^-01^
TcSNP148	3.8E^-01^	6.0E^-01^
TcSNP150	4.2E^-01^	6.4E^-01^
TcSNP154	3.9E^-01^	6.1E^-01^
TcSNP173	4.3E^-01^	6.5E^-01^
TcSNP226	6.4E^-01^	8.1E^-01^
TcSNP230	6.3E^-01^	7.9E^-01^
TcSNP242	4.4E^-01^	6.6E^-01^
TcSNP281	7.3E^-01^	8.6E^-01^
TcSNP290	4.7E^-01^	6.8E^-01^
TcSNP309	4.4E^-01^	6.6E^-01^
TcSNP339	4.8E^-01^	6.9E^-01^
TcSNP341	3.9E^-01^	6.1E^-01^
TcSNP363	4.3E^-01^	6.5E^-01^
TcSNP372	4.2E^-01^	6.4E^-01^
TcSNP380	3.9E^-01^	6.1E^-01^
TcSNP414	3.8E^-01^	6.0E^-01^
TcSNP429	4.7E^-01^	6.9E^-01^
TcSNP519	4.0E^-01^	6.3E^-01^
TcSNP522	4.1E^-01^	6.3E^-01^
TcSNP534	3.8E^-01^	6.0E^-01^
TcSNP546	3.8E^-01^	6.0E^-01^
TcSNP560	3.8E^-01^	6.0E^-01^
TcSNP577	4.0E^-01^	6.2E^-01^
TcSNP591	3.8E^-01^	5.9E^-01^
TcSNP619	4.1E^-01^	6.3E^-01^
TcSNP636	4.1E^-01^	6.3E^-01^
TcSNP642	4.4E^-01^	6.6E^-01^
TcSNP645	4.5E^-01^	6.7E^-01^
TcSNP703	3.8E^-01^	6.1E^-01^
TcSNP723	6.6E^-01^	8.1E^-01^
TcSNP953	4.2E^-01^	6.4E^-01^
TcSNP994	4.1E^-01^	6.4E^-01^
TcSNP1060	5.9E^-01^	7.7E^-01^
Tcm001s08353112	3.8E^-01^	5.9E^-01^
Tcm001s36937466	4.4E^-01^	6.6E^-01^
Tcm001s37350335	3.8E^-01^	6.0E^-01^
Tcm002s00846551	4.1E^-01^	6.3E^-01^
Tcm002s07831310	3.8E^-01^	5.9E^-01^
Tcm002s10122518	3.8E^-01^	6.0E^-01^
Tcm002s34592845	4.6E^-01^	6.8E^-01^
Tcm002s35727800	4.4E^-01^	6.6E^-01^
Tcm003s32803814	4.3E^-01^	6.5E^-01^
Tcm004s02695191	3.8E^-01^	6.0E^-01^
Tcm004s04980962	3.8E^-01^	6.0E^-01^
Tcm004s20311304	3.9E^-01^	6.1E^-01^
Tcm004s23094642	5.3E^-01^	7.3E^-01^
Tcm004s25603617	4.0E^-01^	6.2E^-01^
Tcm005s05086606	4.2E^-01^	6.4E^-01^
Tcm005s07951625	3.8E^-01^	6.0E^-01^
Tcm005s30523742	4.4E^-01^	6.6E^-01^
Tcm005s31040891	3.8E^-01^	6.0E^-01^
Tcm005s31319407	3.8E^-01^	6.0E^-01^
Tcm005s38962849	3.9E^-01^	6.1E^-01^
Tcm006s16820907	4.0E^-01^	6.2E^-01^
Tcm006s22513846	4.3E^-01^	6.5E^-01^
Tcm006s25228227	3.8E^-01^	6.0E^-01^
Tcm006s25344190	3.9E^-01^	6.2E^-01^
Tcm007s00632521	3.8E^-01^	6.0E^-01^
Tcm007s04235015	3.9E^-01^	6.1E^-01^
Tcm007s04402504	3.8E^-01^	6.0E^-01^
Tcm007s04810899	3.8E^-01^	6.0E^-01^
Tcm008s00343038	4.0E^-01^	6.2E^-01^
Tcm008s00740874	3.8E^-01^	6.0E^-01^
Tcm008s03821013	4.5E^-01^	6.7E^-01^
Tcm008s05744556	4.1E^-01^	6.3E^-01^
Tcm009s03673797	3.9E^-01^	6.1E^-01^
Tcm009s29674603	3.8E^-01^	6.0E^-01^
Tcm010s02045420	4.4E^-01^	6.6E^-01^
Tcm010s03831523	4.1E^-01^	6.3E^-01^
**TOTAL**	**1.479E^-29^**	**1.203E^-15^**

PID, Probability of Identity for increasing locus combinations; PID-sib, Probability of Identity among siblings for increasing locus combinations.

The results of pairwise multilocus matching showed that a total of 172 groups with 577 samples had intra-plot mislabeling (12.4% of the total samples), demonstrating a medium rate within the collections (**Supplementary Table 5**). In addition, the results revealed a total of 406 synonymous groups involving 2,472 samples (53.1% of the total) that were identified as duplicates in the AGROSAVIA germplasm banks. Palmira had the highest number of duplicates (1,366), while La Suiza had 1,106 duplicates. The number of duplicated individuals within each group ranged from 2 to 264, with 134 groups consisting of two individuals each. Detailed information about the identified duplicates is listed in **Supplementary Table 6**.

### 3.3 Analysis of population structure and detection of diversity gaps in the collection

**Figure 3:**
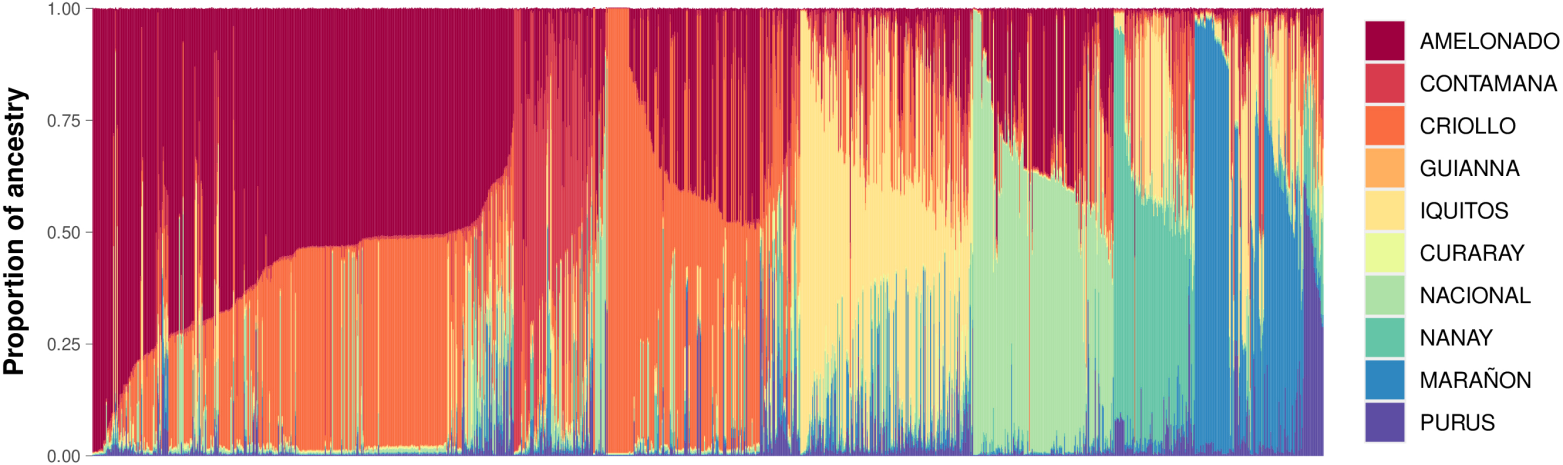
Population structure of the cacao collection from AGROSAVIA. Supervised analysis using the 10 reference cacao populations. Each bar corresponds to an individual, and the height corresponds to the proportion of ancestry explained.

Among the 4,653 cacao samples analyzed, all ten known reference genetic groups were identified. However, only six were predominant within the AGROSAVIA collection. Most accessions exhibited admixture ancestry, with the following distribution: Amelonado (28.6%), Criollo (22.6%), Iquitos (13%), Nacional (10.2%), Contamana (7.2%), Marañon (7.1%), Nanay (5.7%), Purus (3.6%), Curaray (1.3%), and Guiana (0.7%) (**Figure 3**). The last four groups were present at notably low levels.

**Figure 4:**
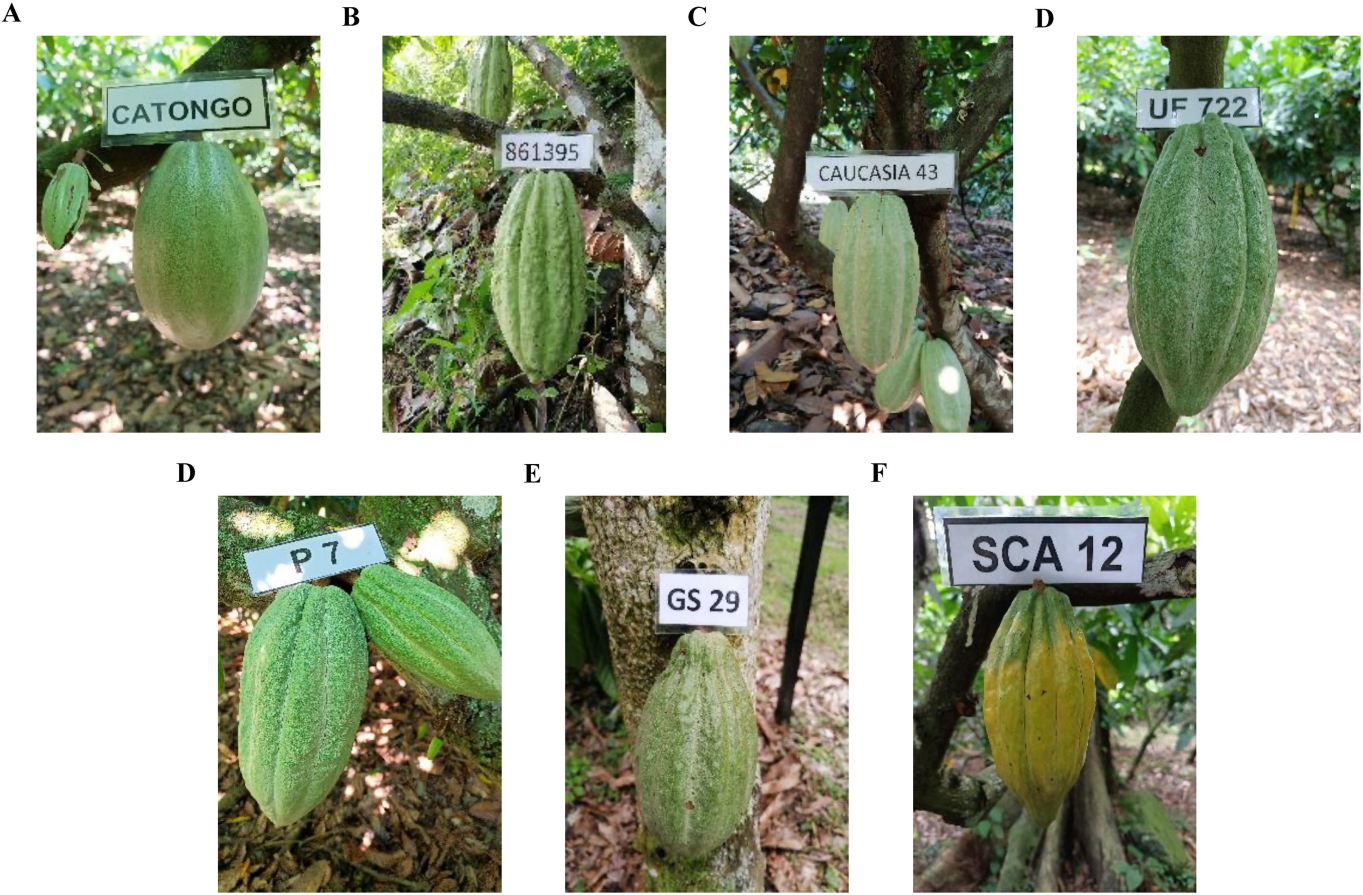
Field representation of seven prominent cacao genetic groups identified in the study. The image illustrates the trees corresponding to each of these groups, showcasing the genetic diversity within the cacao population. **(A)** Amelonado, **(B)** Criollo, **(C)** Iquitos, **(D)** Nacional, **(E)** Nanay, **(F)** Marañon, and **(G)** Contamana.

**Figure 4** illustrates the cacao trees belonging to the seven most representative genetic groups observed in our study. Each group, selected for its distinct genetic characteristics, is displayed within the field setting to emphasize the diversity of the cacao population across the studied regions. The visual representation of these genetic groups allows for a clearer understanding of their spatial distribution and potential implications for cacao breeding and conservation efforts.

### 3.4 Core collections

Using the Core Hunter 3 software, core collections were determined from an initial dataset of 950 cacao samples for La Suiza and 1,223 cacao samples for Palmira. The resulting core subset consisted of 190 and 246 samples, respectively, that captured the full genetic variability present in the original population (**Supplementary Table 7**). All diversity indices were higher compared to the original collections even when duplicates were removed (**Table 1**). The I index showed mean values of 0.619 for Palmira core collection and 0.606 for La Suiza. The H_O_ averaged 0.418 and 0.421, while H_E_ was 0.431 and 0.419, for Palmira and La Suiza core collections, respectively.

## 4 Discussion

### 4.1 Challenges in cacao conservation

Historically, cacao has benefited from an extensive exchange of germplasm material between genebanks across regions worldwide and within countries (Gopaulchan et al., 2019; Rodriguez-Medina et al., 2019). This movement of germplasm has facilitated the domestication of the species, particularly in South America, likely driven by cultural interactions between the Amazon and the Pacific coast over 5,000 years. The development of several landraces shaped by human activity allowed hybridization between cultivars and wild relatives, favoring the adaptation of *T. cacao* to new environments and shaping the genetic basis of the present-day cacao (Lanaud et al., 2024). While hybridization still occurs naturally and through controlled breeding programs, cacao germplasm is maintained by vegetative propagation under field conditions (Rodriguez-Medina et al., 2019). However, the continued exchange of plant materials, often conducted without standardized identifiers, consistent labeling, or complete metadata, represents ongoing challenges for accurate accession tracking (Singh et al., 2019). Consequently, new accessions often lack accurate information regarding their origin. This issue is not unique to cacao and affects numerous germplasm banks worldwide, highlighting the urgent need to improve identity management systems (Bakkali et al., 2019; Migicovsky et al., 2019; de Oliveira et al., 2020). The significance of our study lies in its contribution to preserving a meticulously categorized collection with minimal genetic redundancy, enhancing accuracy, and reducing maintenance costs (Panis et al., 2020). Additionally, our findings emphasize the importance for breeders to understand the molecular diversity within germplasm collections. Such understanding can assist breeding strategies aimed at developing improved new cultivars with higher yields, better quality attributes, and greater resilience to both biotic and abiotic stresses.

### 4.2 Genetic diversity assessment

The required number of markers for accurately identifying cacao germplasm and assessing genetic diversity within a genebank depends on the species’ genetic variability, sample size, and the specific molecular marker used (Alhasnawi et al., 2024). In species like *T. cacao*, which exhibit significant genetic diversity, it is essential to use a substantial number of markers spread across the genome to effectively detect allelic variations. In the present study, we selected a set of 96 SNP markers that have been used in previous studies (Lukman et al., 2014; Cosme et al., 2016; Lindo et al., 2018; Wang et al., 2020; Dillon et al., 2024). After applying quality control filters, 77 SNPs were retained for analysis, from which we achieved a robust representation of the genetic groups identified for cacao and obtained diversity values comparable to studies that used a larger number of SNPs (Osorio-Guarín et al., 2020). Regardless of the marker type or species diversity, it is crucial to determine the optimal number of markers needed to assess the genetic diversity and accession identification. In our study, the genotype accumulation curve used to establish this optimal number indicated that the selected markers were adequate for distinguishing the cacao accessions, revealing 2,630 unique MLGs among the 4,653 samples.

This study genotyped the largest number of cacao samples to date, with 4,653 cacao trees representing the entire cacao collections maintained in AGROSAVIA. The H_O_ value is comparable or higher than previous SNP-based diversity assessments reported for cacao (Ji et al., 2013; Takrama et al., 2014; Cosme et al., 2016). The genetic diversity values indicated that both collections, from Palmira and La Suiza, are very similar in their diversity. The Palmira germplasm collection is more diverse in H_E_, possibly due to the highest number of individuals (2,597) compared to the La Suiza germplasm bank (2,056 individuals) and because the two collections differ in their working cacao accessions. However, they conserved mainly the same accessions because La Suiza germplasm bank originated as a copy of the Palmira germplasm bank.

Other studies on cacao using KASP technology found similar heterozygosity values. Olasupo et al. (2018) found H_O_ = 0.248 and H_E_ = 0.413 using 63 SNPs across 1,457 samples from Nigeria’s cacao breeding programs. They also detected a high rate of mislabeling among recently introduced international germplasm in Nigeria. Bhattacharjee et al. (2023), analyzed 376 samples with 20 SNPs using KASP and observed lower H_O_ and H_E_ values compared to our study. However, they also reported high mislabeling rates in African countries like Sierra Leone (33.33%) and Togo (45.45%), which is comparable to our findings.

### 4.3 Diversity gaps in Colombian cacao collections

The present results suggested that the Colombian cacao collections have a high level of allelic diversity, as measured by heterozygosity and AMOVA. However, a gap in genetic diversity was detected, particularly in terms of the underrepresentation of known Amazon cacao populations. In addition, approximately 30% of the accessions in the collection belong to the Trinitario type, which is significantly over-represented and exhibits a high level of genetic redundancy due to the presence of duplicates. The analysis also revealed mislabeling in the introduced international genotypes. Several international accessions labeled as Parinari, Scavina, and IMC were not genetically aligned with their expected reference groups, indicating discrepancies in their identity. Similar patterns were observed for collections in both locations, confirming the similarity between Palmira and La Suiza germplasm banks. Therefore, the La Suiza collection can be considered a backup or reserve collection for Palmira. This finding is further supported by PCA analysis, which shows similar clustering patterns for both collections, with differences potentially explained by the specific cultivars developed by each respective plant breeding program.

The AMOVA results indicated that the majority of genetic variation occurs within collections rather than between them, which is expected given the presence of shared accessions. Although maintaining duplicate collections might seem cost-inefficient, preserving backup germplasm at two distinct sites offers significant advantages. The contrasting environmental conditions in Palmira and La Suiza allow for the evaluation of the same materials under varying climatic and disease pressures.

Notably, Palmira and La Suiza differ in their genetic composition. Palmira holds a greater number of accessions with mixed ancestry compared to the La Suiza genebank. In contrast, La Suiza contains fewer accessions with Amelonado and Iquitos ancestry, although both genebanks maintain comparable numbers of Contamana and Marañon ancestry accessions. Palmira’s broader diversity, particularly in mixed ancestries, provides a wider genetic base for selecting traits such as disease resistance and yield. Meanwhile, La Suiza’s narrower diversity may limit genetic variability but could represent a more focused genetic pool ideal for specific breeding objectives. The germplasm conserved at La Suiza has been evaluated for several agronomic traits, including disease resistance and productivity (Osorio-Guarín et al., 2020). These genetic distinctions between the two genebanks offer a rich and complementary spectrum of resources for breeding efforts.

The genetic resources maintained in national genebanks have been effectively utilized to develop and release local cocoa cultivars adapted to Colombia’s diverse agroecological conditions. AGROSAVIA conducted regional agronomic evaluations of eight selected cacao genotypes across four sites in the Montaña Santandereana and Magdalena Medio subregions. Among these, TCS 13 and TCS 19 demonstrated superior physiological and agronomic performance, including high photosynthetic capacity, improved water-use efficiency, resistance to moniliasis, and self-compatibility. These traits contributed to high yields (1.5–1.8 kg tree¹ year¹) under agroforestry systems, leading to their registration as commercial cultivars in the Colombian National Register in 2017 (Agudelo-Castañeda et al., 2017, 2018, 2023). In addition, AGROSAVIA released in 2014, the cultivars TCS 01, characterized by high self-compatibility and robust yields, and TCS 06, which has strong resistance to FPRD (Suárez et al., 2022). Finally, this dual-site conservation and breeding approach provides a solid foundation for advanced breeding strategies, including genomic selection. It also supports the application of the Focused Identification of Germplasm Strategy (FIGS), which leverages environmental and passport data to identify germplasm with desirable traits. The existence of duplicate collections in distinct environments can enhance the effectiveness of FIGS by providing comparative data sets that improve the accuracy of trait prediction and targeted germplasm mining (Sunitha et al., 2024).

The genetic groups identified using the 77 SNPs panel showed concordance with those previously studied using SSR markers by Motamayor et al. (2008), demonstrating the usefulness of this SNP panel for assessing genetic diversity and population structure in cacao. However, the population structure analysis revealed that the genetic groups Nanay, Curaray, Purus, and Guiana are underrepresented in AGROSAVIA’s collections. The limited number of accessions from these groups belonging to the Amazon genetic groups within the collection fails to capture the comprehensive intra-population genetic diversity of *T. cacao*. To address this gap, it is imperative to introduce new accessions from international germplasm banks belonging to these specific genetic groups. It is important to note that population assignment in this study was analyzed using the framework of 10 genetic cacao populations (Motamayor et al., 2008). As a result, the analysis was unable to detect accessions that may represent new genetic groups. Such undetected diversity has been highlighted in recent expeditions and studies (Osorio-Guarín et al., 2017; Argout et al., 2023). The population structure of this collection will be further analyzed, including recently collected wild cacao (Argout et al., 2023; Zhang and Motilal, 2016) as reference.

### 4.4 Addressing mislabeling and redundancy in germplasm collections

The present results highlighted the need for corrective actions to address mislabeling within the AGROSAVIA cacao collection. Using a plot heterogeneity strategy, we assessed the occurrence of mixed genotypes within field plots and identified a high incidence of mislabeling, defined as two distinct accessions sharing identical SNP patterns. In addition, we identified a high rate of duplicate accessions that must be addressed to reduce genetic redundancy within the collection. The problem of multiple individuals having the same genotype (SNP pattern) at all loci can be addressed by increasing the number of loci in a study, thereby decreasing the probability that two individuals have the same MGL. We used an MLG strategy along with the PID, a measure that two individuals in a population share the same multilocus genotype. PID depends on the number of loci analyzed, the level of allelic diversity, and the degree of relatedness among individuals in the population. To minimize the potential errors in estimating PID, we used the PID-sib metric, which provides a conservative upper bound for determining the number of loci necessary to reliably distinguish individuals (Waits et al., 2001). In this study, both values, PID and PID-sib, demonstrated the ability of the SNP markers to discriminate individuals. Notably, our PID value (1.479 x 10^-29^) was significantly higher than the PID reported by Olasupo et al. (2018), who obtained a value on the order of 10^−6^. This result demonstrates the good resolution of our 77 SNP panel to verify the true-to-type trees.

Our results highlight a critical issue of genetic misidentification and redundancy that requires immediate attention. Such inconsistencies may originate from errors during the initial establishment of the genebank or the introduction of new accessions. Mislabeling is a common problem in clonal propagation, where seedlings may be mistakenly transplanted instead of true-to-type clones, especially accessions acquired from older expeditions (Turnbull et al., 2003; Motilal et al., 2013). Additionally, planting or grafting incorrect accessions in field plots could contribute to the loss of genetic integrity. In many cases, material was collected in the form of pods (seeds) rather than vegetative cuttings, increasing the likelihood of confusion due to regional variations in accession names.

To improve the accuracy and management of field collections, we recommend permanent labels using quick response (QR) along with routine analysis of molecular and phenotypic data. These steps are essential to ensure the long-term reliability and functionality of the gene bank, and their successful implementation will require close collaboration of germplasm curators. The high rate of intra-accession mislabeling and genetic redundancy found in AGROSAVIA germplasm establishes a strong foundation for revisiting and correcting existing records. Other strategy to address this issue is the conformation of core collections. In fact, we found an increase in diversity indices of defined core collections highlighting its effectiveness in enriching genetic variation while reducing redundancy. This improvement is particularly valuable for cacao breeding programs, as it ensures a smaller, more manageable subset that can be evaluated for agronomical traits retaining the genetic breadth of the original collection. These core collections could provide a robust foundation for selecting parent lines with desirable traits and enhance the potential for genetic gains through both conventional and molecular breeding approaches (Gu et al., 2023).

## 5 Conclusions

The SNP variants used in this study proved to be highly informative and demonstrated strong discriminatory power. The results revealed high molecular diversity and low genetic differentiation between the two germplasm banks, which is expected given that both Palmira and La Suiza conserve the same collection. These findings have important implications for cacao breeding, as they provide insights into which genetic ancestries are well represented (Amelonado, Criollo, Contamana, Nacional, Marañon, and Iquitos) and which are underrepresented (Purus, Curaray, Nanay, and Guiana). This information supports the development of targeted conservation strategies to preserve rare alleles and enables the design of crossbreeding programs aimed to exploit heterosis and to generate novel allele combinations to improve traits such as yield, quality, and disease resistance.

The detection of mislabeled accessions is particularly valuable, as it helps ensure the accuracy of genetic identities and prevents errors in breeding, conservation, and research activities. Likewise, the identification of clonal duplicates contributes to the efficient management of germplasm banks by reducing redundancy and focusing resources to maintain genetically unique accessions. To further enhance the genetic variability within the germplasm collection, we recommend implementing strategies such as investing in pre-breeding efforts, expanding the geographic range of germplasm introductions, and collecting wild material. These approaches will be critical to maximizing the potential for genetic improvement and ensuring the long-term sustainability of cacao breeding efforts in the region.

## Data Availability

The original contributions presented in the study are included in the article/**Supplementary Material**. Further inquiries can be directed to the corresponding authors.

## References

[B1] Agudelo-Castañeda G. A. Antolinez-Sandoval E. Y. Báez-Daza E. Y. Jaimes-Suárez Y. Y. Romero-Guerrero G. A. (2023). Nuevas variedades de cacao seleccionadas en Colombia. Rev. Mex Cienc Agric. 14, 315–326. doi: 10.29312/remexca.v14i3.3057

[B2] Agudelo-Castañeda G. A. Cadena-Torres J. Almanza-Merchán P. J. Pinzón-Sandoval E. H. (2018). Desempeño fisiológico de nueve genotipos de cacao (*Theobroma cacao* L.) bajo la sombra de tres especies forestales en Santander, Colombia. Rev. Colombiana Cienc. Hortícolas 12, 223–232. doi: 10.17584/rcch.2018v12i1.7341

[B3] Agudelo-Castañeda G. Calderón G. Antolinez Sandoval E. Baez E. (2017). Nuevas variedades de cacao TCS (Theobroma Corpoica La Suiza) 13 y 19. doi: 10.13140/RG.2.2.19293.28642

[B4] Aikpokpodion P. O. Kolesnikova-Allen M. Adetimirin V. O. Guiltinan M. J. Eskes A. B. Motamayor J.-C.. (2010). Population structure and molecular characterization of Nigerian field genebank collections of cacao, *Theobroma cacao* L. Silvae Genet. 59, 273–285. doi: 10.1515/sg-2010-0039

[B5] Alhasnawi A. Alasadiy Y. Doni F. (2024). Assessment of the genetic diversity in plants using molecular markers: a review and perspective. Trop. Agric. 101, 120–134. doi: 0041–3216/2024/010120–134

[B6] Allegre M. Argout X. Boccara M. Fouet O. Roguet Y. Bérard A.. (2012). Discovery and mapping of a new expressed sequence tag-single nucleotide polymorphism and simple sequence repeat panel for large-scale genetic studies and breeding of *Theobroma cacao* L. DNA Res. 19, 23–35. doi: 10.1093/dnares/dsr039 22210604 PMC3276266

[B7] Arévalo-Gardini E. Arévalo-Gardini C. Meinhardt L. Motilal L. Umaharan P. Sankar A.. (2023). “Wild cacao in Peruvian Amazon - Progress in analysis of genetic diversity and population structure,” in Plant and Animal Genome Conference (PAG 30), Cacao Genomics Workshop, , San Diego, CA.

[B8] Argout X. Droc G. Fouet O. Rouard M. Labadie K. Rhoné B.. (2023). Pangenomic exploration of *Theobroma cacao*: New Insights into Gene Content Diversity and Selection During Domestication. bioRxiv, 2023.11.03.565324. doi: 10.1101/2023.11.03.565324

[B9] Asare Bediako K. Padi F. K. Obeng-Bio E. Ofori A. (2025). Genetic diversity and parentage of cacao ( *Theobroma cacao* L.) populations from Ghana using single nucleotide polymorphism (SNP) markers. Plant Genet. Resour. 23, 40–47. doi: 10.1017/S1479262124000510

[B10] Bakkali A. Essalouh L. Tollon C. Rivallan R. Mournet P. Moukhli A.. (2019). Characterization of Worldwide Olive Germplasm Banks of Marrakech (Morocco) and Córdoba (Spain): Towards management and use of olive germplasm in breeding programs. PloS One 14, e0223716. doi: 10.1371/JOURNAL.PONE.0223716 31622375 PMC6797134

[B11] Ballesteros W. Lagos T. C. (2016). Morphological characterization of elite cacao trees ( *Theobroma cacao* L.) in Tumaco, Nariño, Colombia. Rev. Colombiana Cienc. Hortícolas 9, 313. doi: 10.17584/rcch.2015v9i2.4187. L, H. F.

[B12] Bartley B. G. D. (2005). The genetic diversity of cacao and its utilization. Ed. Bartley B. G. D. (UK: CABI Publishing). doi: 10.1079/9780851996196.0000

[B13] Bhattacharjee R. Luseni M. M. Ametefe K. Agre P. A. Kumar P. L. Grenville-Briggs L. J. (2023). Genetic diversity and population structure of cacao (*Theobroma cacao* L.) germplasm from Sierra Leone and Togo based on KASP- SNP genotyping. Agronomy 14, 2458. doi: 10.3390/agronomy14112458

[B14] Borrone J. W. Brown J. S. Kuhn D. N. Motamayor J. C. Schnell R. J. (2007). Microsatellite markers developed from *Theobroma cacao* L. expressed sequence tags. Mol. Ecol. Notes 7, 236–239. doi: 10.1111/j.1471-8286.2006.01561.x

[B15] Bunjkar A. Walia P. Sandal S. S. (2024). Unlocking genetic diversity and germplasm characterization with molecular markers: strategies for crop improvement. J. Adv. Biol. Biotechnol. 27, 160–173. doi: 10.9734/jabb/2024/v27i6873

[B16] Cosme S. Cuevas H. E. Zhang D. Oleksyk T. K. Irish B. M. (2016). Genetic diversity of naturalized cacao ( *Theobroma cacao* L.) in Puerto Rico. Tree Genet. Genomes 12, 88. doi: 10.1007/s11295-016-1045-4

[B17] Cuppen E. (2007). Genotyping by allele-specific amplification (KASPar). CSH Protoc. 2007, pdb.prot4841–pdb.prot4841. doi: 10.1101/PDB.PROT4841 21357174

[B18] Daymond A. Bekele F. (2022). “Cacao,” in Cash Crops: Genetic Diversity, Erosion, Conservation and Utilization. Eds. Priyadarshan P. M. Jain S. M. (Springer International Publishing, Cham), 23–53. doi: 10.1007/978-3-030-74926-2_2

[B19] De Beukelaer H. Davenport G. F. Fack V. (2018). Core Hunter 3: Flexible core subset selection. BMC Bioinf. 19, 1–12. doi: 10.1186/S12859-018-2209-Z/TABLES/3 PMC6092719 29855322

[B20] de Oliveira G. L. de Souza A. P. de Oliveira F. A. Zucchi M. I. de Souza L. M. Moura M. F. (2020). Genetic structure and molecular diversity of Brazilian grapevine germplasm: Management and use in breeding programs. PloS One 15, e0240665. doi: 10.1371/JOURNAL.PONE.0240665 33057449 PMC7561202

[B21] Dillon N. L. Zhang D. Nauheimer L. Toramo E. Nagalevu P. Melteras M. V.. (2024). Understanding the cocoa genetic resources in the Pacific to assist producers to supply the growing craft market. N Z J. Crop Hortic. Sci. 52, 306–320. doi: 10.1080/01140671.2023.2278788

[B22] Gopaulchan D. Motilal L. A. Bekele F. L. Clause S. Ariko J. O. Ejang H. P.. (2019). Morphological and genetic diversity of cacao ( *Theobroma cacao* L.) in Uganda. Physiol. Mol. Biol. Plants 25, 361–375. doi: 10.1007/s12298-018-0632-2 30956420 PMC6419697

[B23] Gu R. Fan S. Wei S. Li J. Zheng S. Liu G. (2023). Developments on core collections of plant genetic resources: do we know enough? Forests 14, 926. doi: 10.3390/F14050926/S1

[B24] Gutiérrez O. A. Martinez K. Zhang D. Livingstone D. S. Turnbull C. J. Motamayor J. C.. (2021). Selecting SNP markers reflecting population origin for cacao ( *Theobroma cacao* L.) germplasm identification. Beverage Plant Res. 1, 1–9. doi: 10.48130/BPR-2021-0015

[B25] ICCO (2024). Statistics. Available online at: https://www.icco.org/statistics/ (Accesed August 24, 2024).

[B26] Irish B. M. Goenaga R. Zhang D. Schnell R. Brown J. S. Motamayor J. C. (2010). Microsatellite fingerprinting of the USDA-ARS tropical agriculture research station cacao ( *Theobroma cacao* L.) germplasm collection. Crop Sci. 50, 656–667. doi: 10.2135/cropsci2009.06.0299

[B27] Ji K. Zhang D. Motilal L. A. Boccara M. Lachenaud P. Meinhardt L. W. (2013). Genetic diversity and parentage in farmer varieties of cacao ( *Theobroma cacao* L.) from Honduras and Nicaragua as revealed by single nucleotide polymorphism (SNP) markers. Genet. Resour Crop Evol. 60, 441–453. doi: 10.1007/s10722-012-9847-1

[B28] Kalinowski S. Manlove K. Taper M. (2007). ONCOR: a computer program for genetic stock identification, v.2. Available online at: http://www.montana.edu/kalinowski/Software/ONCOR.htm (Accessed March 13, 2024).

[B29] Kamvar Z. N. Tabima J. F. Grünwald N. J. (2014). Poppr: an R package for genetic analysis of populations with clonal, partially clonal, and/or sexual reproduction. PeerJ 2, e281. doi: 10.7717/peerj.281 24688859 PMC3961149

[B30] Kodoth N. (2021). “Cacao,” in Tree Crops: Harvesting Cash from the World’s Important Cash Crops (Springer International Publishing, Cham, Switzerland), 153–210. doi: 10.1007/978-3-030-62140-7

[B31] Kongor J. E. Owusu M. Oduro-Yeboah C. (2024). Cocoa production in the 2020s: challenges and solutions. CABI Agric. Bioscience 5, 1–28. doi: 10.1186/S43170-024-00310-6

[B32] Lanaud C. Vignes H. Utge J. Valette G. Rhoné B. Garcia Caputi M.. (2024). A revisited history of cacao domestication in pre-Columbian times revealed by archaeogenomic approaches. Sci. Rep. 14, 1–16. doi: 10.1038/s41598-024-53010-6 38453955 PMC10920634

[B33] Li Y. Zhang D. Motilal L. A. Lachenaud P. Mischke S. Meinhardt L. W. (2021). Traditional varieties of cacao ( *Theobroma cacao*) in Madagascar: their origin and dispersal revealed by SNP markers. Beverage Plant Res. 1, 1–7. doi: 10.48130/BPR-2021-0004

[B34] Lindo A. A. Robinson D. E. Tennant P. F. Meinhardt L. W. Zhang D. (2018). Molecular characterization of cacao ( *Theobroma cacao*) germplasm from Jamaica using single nucleotide polymorphism (SNP) markers. Trop. Plant Biol. 11, 93–106. doi: 10.1007/S12042-018-9203-5/METRICS

[B35] Livingstone D. S. Motamayor J. C. Schnell R. J. Cariaga K. Freeman B. Meerow A. W.. (2011). Development of single nucleotide polymorphism markers in *Theobroma cacao* and comparison to simple sequence repeat markers for genotyping of Cameroon clones. Mol. Breed. 27, 93–106. doi: 10.1007/s11032-010-9416-2

[B36] López-Hernández M. del P. Sandoval-Aldana A. P. García-Lozano J. Criollo-Nuñez J. (2021). Estudio morfoagronómico de materiales de cacao ( *Theobroma cacao* L.) de diferentes zonas productoras en Colombia. Ciencia y Agricultura 18, 98–109. doi: 10.19053/01228420.v18.n3.2021.12570

[B37] Lukman Zhang D. Susilo A. W. Dinarti D. Bailey B. Mischke S.. (2014). Genetic identity, ancestry and parentage in farmer selections of cacao from aceh, Indonesia revealed by single nucleotide polymorphism (SNP) markers. Trop. Plant Biol. 7, 133–143. doi: 10.1007/s12042-014-9144-6

[B38] Mahabir A. Motilal L. A. Gopaulchan D. Ramkissoon S. Sankar A. Umaharan P. (2020). Development of a core SNP panel for cacao ( *Theobroma cacao* L.) identity analysis. Genome 63, 103–114. doi: 10.1139/gen-2019-0071 31682479

[B39] Mahabir A. Motilal L. A. Gopaulchan D. Sankar A. Umaharan P. (2017). “Identification of a core SNP panel for cacao identity and population analyses,” in International Symposium on Cocoa Research (ISCR), Lima, Peru, 13–17 November 2017 Identification, Vol. 1. 2.

[B40] Mammadov J. Aggarwal R. Buyyarapu R. Kumpatla S. (2012). SNP markers and their impact on plant breeding. Int. J. Plant Genomics 2012, 1–10. doi: 10.1155/2012/728398 PMC3536327 23316221

[B41] Migicovsky Z. Warschefsky E. Klein L. L. Miller A. J. Migicovsky Z. Warschefsky E.. (2019). Using living germplasm collections to characterize, improve, and conserve woody perennials. Crop Sci. 59, 2365–2380. doi: 10.2135/CROPSCI2019.05.0353

[B42] Motamayor J. C. Lachenaud P. Wallace J. Loor R. Kuhn D. N. Brown S.. (2008). Geographic and genetic population differentiation of the Amazonian chocolate tree ( *Theobroma cacao* L.). PloS One 3, 1–8. doi: 10.1371/journal.pone.0003311 PMC2551746 18827930

[B43] Motilal L. A. Zhang D. Mischke S. Meinhardt L. W. Umaharan P. (2013). Microsatellite-aided detection of genetic redundancy improves management of the International Cocoa Genebank, Trinidad. Tree Genet. Genomes 9, 1395–1411. doi: 10.1007/S11295-013-0645-5/METRICS

[B44] Olasupo F. O. Adewale D. B. Aikpokpodion P. O. Muyiwa A. A. Bhattacharjee R. Gutierrez O. A.. (2018). Genetic identity and diversity of Nigerian cacao genebank collections verified by single nucleotide polymorphisms (SNPs): a guide to field genebank management and utilization. Tree Genet. Genomes 14, 1–16. doi: 10.1007/S11295-018-1244-2/FIGURES/3

[B45] Osorio-Guarín J. A. Berdugo-Cely J. Coronado R. A. Zapata Y. P. Quintero C. Gallego-Sánchez G.. (2017). Colombia a source of cacao genetic diversity as revealed by the population structure analysis of germplasm bank of *Theobroma cacao* L. Front. Plant Sci. 8. doi: 10.3389/fpls.2017.01994 PMC5702303 29209353

[B46] Osorio-Guarín J. A. Berdugo-Cely J. A. Coronado-Silva R. A. Baez E. Jaimes Y. Yockteng R. (2020). Genome-wide association study reveals novel candidate genes associated with productivity and disease resistance to Moniliophthora spp. in cacao ( *Theobroma cacao* L.). G3: Genes Genomes Genet. 10, 1713–1725. doi: 10.1534/g3.120.401153 PMC7202020 32169867

[B47] Padi F. K. Ofori A. Takrama J. Djan E. Opoku S. Y. Dadzie A. M.. (2015). The impact of SNP fingerprinting and parentage analysis on the effectiveness of variety recommendations in cacao. Tree Genet. Genomes 11, 1–14. doi: 10.1007/S11295-015-0875-9/METRICS

[B48] Panis B. Nagel M. den Houwe I. V. (2020). Challenges and prospects for the conservation of crop genetic resources in field genebanks, in *in vitro* collections and/or in liquid nitrogen. Plants 9, 1–22. doi: 10.3390/PLANTS9121634 PMC7761154 33255385

[B49] Peakall R. Smouse P. (2006). genalex 6: genetic analysis in Excel. Population genetic software for teaching and research. Mol. Ecol. Notes 6, 288–295. doi: 10.1111/j.1471-8286.2005.01155.x PMC3463245 22820204

[B50] Peakall R. Smouse P. (2012). GenAlEx 6.5: Genetic analysis in Excel. Population genetic software for teaching and research an update. Bioinformatics 1, 6–8. doi: 10.1093/bioinformatics/bts460 PMC3463245 22820204

[B51] Pritchard J. K. Stephens M. Donnelly P. (2000). Inference of population structure using multilocus genotype data. Genetics 155, 945–959. doi: 10.1093/genetics/155.2.945 10835412 PMC1461096

[B52] Ramos Ospino A. Gómez Alvaréz M. MaChado-Sierra E. Aranguren Y. (2020). Caracterización fenotípica y genotípica de cultivares de cacao (*Theobroma cacao* L.) de Dibulla, La Guajira, Colombia. Ciencia Tecnología Agropecuaria 21, 1–17. doi: 10.21930/rcta.vol21_num3_art:1557

[B53] Rodriguez-Medina C. Arana A. C. Sounigo O. Argout X. Alvarado G. A. Yockteng R. (2019). Cacao breeding in Colombia, past, present and future. Breed Sci. 69, 373–382. doi: 10.1270/jsbbs.19011 31598069 PMC6776146

[B54] Rodríguez-Medina C. Sounigo O. Yockteng Benalcázar R. Romero Guerrero G. A. Monsalve García D. A. (2023). Programa de mejoramiento genético de cacao en Colombia: una propuesta para aunar esfuerzos a nivel nacional en beneficio del productor de cacao Colombiano. Ed. Gaona García L. (Mosquera, Colombia: Corporación Colombiana de Investigación Agropecuaria (agrosavia).

[B55] Semagn K. Babu R. Hearne S. Olsen M. (2014). Single nucleotide polymorphism genotyping using Kompetitive Allele Specific PCR (KASP): Overview of the technology and its application in crop improvement. Mol. Breed. 33, 1–14. doi: 10.1007/S11032-013-9917-X

[B56] Singh N. Wu S. Raupp W. J. Sehgal S. Arora S. Tiwari V.. (2019). Efficient curation of genebanks using next generation sequencing reveals substantial duplication of germplasm accessions. Sci. Rep. 9, 1–10. doi: 10.1038/s41598-018-37269-0 30679756 PMC6346010

[B57] Suárez Y. Y. J. Castañeda G. A. A. Daza E. Y. B. Bustos F. M. Estrada G. A. R. Molina J. R. (2022). Modelo productivo para el cultivo de cacao ( Theobroma cacao L.) en el departamento de Santander. 2a edición (Mosquera, Colombia: AGROSAVIA).

[B58] Sunitha N. C. Prathibha M. D. Thribhuvan R. Lokeshkumar B. M. Basavaraj P. S. Lohithaswa H. C.. (2024). Focused identification of germplasm strategy (FIGS): a strategic approach for trait-enhanced pre-breeding. Genet. Resour Crop Evol. 71, 1–16. doi: 10.1007/S10722-023-01669-7/METRICS

[B59] Takrama J. Kun J. Meinhardt L. Mischke S. Opoku S. Y. Padi F. K.. (2014). Verification of genetic identity of introduced cacao germplasm in Ghana using single nucleotide polymorphism (SNP) markers. Afr J. Biotechnol. 13, 2127–2136. doi: 10.5897/AJB2013.13331

[B60] Temesgen T. Zigale S. Tamirat B. (2021). Multi environments and genetic-environmental interaction (GxE) in plant breeding and its challenges: A review article. Int. J. Res. Stud. Agric. Sci. 7, 10–18. doi: 10.20431/2454-6224.0704002

[B61] Tripodi P. (2023). The evolution of molecular genotyping in plant breeding. Agronomy 13, 2569. doi: 10.3390/AGRONOMY13102569

[B62] Turnbull C. Butler D. Cryer N. Zhang D. Lanaud C. Daymond A.. (2003). Tackling mislabelling in cocoa germplasm collections. INGENIC Newslett. 8, 8–11.

[B63] van Hintum T. J. L. (2000). Duplication within and between germplasm collections. III. A quantitative model. Genet. Resour Crop Evol. 47, 507–513. doi: 10.1023/A:1008703031415

[B64] Van Treuren R. Van Hintum T. J. L. (2005). The genetic diversity of cacao and its utilization. Ed. Bartley B. G. D. (UK: CABI Publishing). doi: 10.1079/9780851996196.0000

[B65] Waits L. P. Luikart G. Taberlet P. (2001). Estimating the probability of identity among genotypes in natural populations: cautions and guidelines. Mol. Ecol. 10, 249–256. doi: 10.1046/J.1365-294X.2001.01185.X 11251803

[B66] Wang B. Motilal L. A. Meinhardt L. W. Yin J. Zhang D. (2020). Molecular characterization of a cacao germplasm collection maintained in yunnan, China using single nucleotide polymorphism (SNP) markers. Trop. Plant Biol. 13, 359–370. doi: 10.1007/s12042-020-09267-y

[B67] Yadesa L. (2022). Review on genetic-environmental interaction (GxE) and its application in crop breeding. Int. J. Res. Agron. 5, 95–101. doi: 10.33545/2618060X.2022.V5.I2B.115

[B68] Zhang D. Arevalo-Gardini E. Gutarra B. Baligar V. Meinhardt L. (2023). “The newly collected wild cacao germplasm from Peruvian amazon and its implication for disease resistance,” in Plant and Animal Genome XXI Conference, San Diego, CA.

[B69] Zhang D. Martínez W. J. Johnson E. S. Somarriba E. Phillips-Mora W. Astorga C.. (2012). Genetic diversity and spatial structure in a new distinct *Theobroma cacao* L. population in Bolivia. Genet. Resour Crop Evol. 59, 239–252. doi: 10.1007/s10722-011-9680-y

[B70] Zhang D. Mischke S. Johnson E. S. Phillips-Mora W. Meinhardt L. (2009). Molecular characterization of an international cacao collection using microsatellite markers. Tree Genet. Genomes 5, 1–10. doi: 10.1007/s11295-008-0163-z

[B71] Zhang D. Motilal L. (2016). “Origin, dispersal, and current global distribution of cacao genetic diversity BT - cacao diseases: a history of old enemies and new encounters,” in Cacao Diseases: A History of Old Enemies and New Encounters. Eds. Bailey B. A. Meinhardt L. W. (Springer International Publishing, Cham), 3–31. doi: 10.1007/978-3-319-24789-2_1

